# Epigenetic Regulation of Immune Responses in Endocrine-Related Cancers and Its Role in Immunotherapy

**DOI:** 10.3390/cancers17203342

**Published:** 2025-10-16

**Authors:** Evren M. Akyuz, Meryem Gultekin, Judith M. Ramage, Ian Spendlove, Andrew M. Jackson, Jason Adhikaree, Anna A. Malecka

**Affiliations:** 1 School of Veterinary Medicine and Sciences, Faculty of Medicine and Health Sciences, University of Nottingham, Nottingham LE12 5RD, UK; mzyea14@exmail.nottingham.ac.uk; 2 Biodiscovery Institute, University of Nottingham, Nottingham NG7 2RD, UK; meryem.gultekin@nottingham.ac.uk (M.G.); judith.ramage@nottingham.ac.uk (J.M.R.); ian.spendlove@nottingham.ac.uk (I.S.); andrew.jackson@nottingham.ac.uk (A.M.J.); jason.adhikaree2@nottingham.ac.uk (J.A.); 3 School of Medicine, Faculty of Medicine and Health Sciences, University of Nottingham, Nottingham NG7 2UH, UK

**Keywords:** breast cancer, prostate cancer, ovarian cancer, thyroid cancer, immunology, immunotherapy, epigenetics, epi-drugs

## Abstract

Endocrine-related cancers are commonly diagnosed worldwide and are a major cause of cancer-related deaths. While current therapies are effective at earlier stages, they often fail in more advanced or recurring tumors due to the development of primary and secondary resistance. Therefore, new therapeutic approaches are urgently needed. In recent years, new therapeutic avenues, namely immunotherapy and epigenetic modulation, have been successfully explored in multiple cancer types. However, this success has not been observed in endocrine-related cancers. These tumors are considered immunologically cold, characterized by a low immunogenicity, scarce infiltration by immune cells, and highly immunosuppressive environment rendering them resistant to immunotherapy. Recent studies demonstrated the ability of epigenetic agents to simultaneously alter the behavior of tumor and immune cells, giving premise to combination approaches using epigenetic modulation to prime cancer for immunotherapy. This review provides an overview of the immune landscape of endocrine-related cancers and its main epigenetic regulators. We discuss current clinical trials investigating the effect of combination endocrine-related immunotherapy and their challenges to successfully treat endocrine-related cancers.

## Simple Summary

Endocrine-related cancers often acquire resistance to current therapies. New therapeutic approaches such as epigenetic modulation and immunotherapy, while promising, so far have delivered little benefit. Recent research suggests that epigenetic mechanisms are key in the regulation of anti-cancer immune responses and play an important role in the therapeutic resistance of endocrine-related cancers. This review provides an overview of the immune landscape of endocrine-related cancers, including breast, prostate, ovarian and thyroid. We discuss epigenetic modulators of anti-cancer immune responses and current clinical trials combining immunotherapy with epigenetic modulation, with the aim of invigorating anti-cancer immune responses.

## 1. Introduction

Endocrine-related cancers (ERCs) are a group of hormonally dependent tumors which arise from various endocrine tissues, including ovarian, breast, thyroid and prostate. ERCs are commonly diagnosed accounting for around 55% of cancer diagnoses worldwide, primarily driven by prostate and breast cancer (BC) [1]. Despite recent progress in diagnosis and treatment, many ERCs remain resistant to therapy, leading to around 22% of all cancer-related deaths [1]. While these diseases target different anatomic sites and have heterogenous diagnosis and treatment, the majority of ERCs display low mutational burden, and an immune-cold phenotype.

To survive and grow, cancer cells require support from other cells in their environment, including infiltrating immune cells. Immune cells have the ability to eradicate cancer cells; however, in the tumor microenvironment (TME), they often become anergic or exhausted and instead promote tumor growth. Cancer immunotherapy, which aims to activate immune responses, achieved significant progress in the treatment of multiple types of cancer, including melanoma and lung cancer [2,3]. However, so far this success has not been observed in ERCs, which utilize various mechanisms to escape immune recognition and elimination, including immune evasion and immune sculpting, by modulating immune cells phenotypes [4,5,6,7]. These modifications are often driven by dynamic modulation of the epigenetic regulation of cancer and immune cells.

Epigenetic mechanisms control the accessibility of genes and their transcription through dynamic and reversible processes. Their dysregulation plays a key role in cancer development and progression and has recently been added to the hallmarks of cancer [8]. In cancer cells, epigenetic dysregulation promotes proliferation, invasion, and metastasis [9]. However, epigenetic modulation affects not only cancer cells but also cells in the TME including infiltrating immune cells, thereby modulating anti-cancer immune responses. Epigenetic reprogramming of immune cells can be triggered by factors secreted by cancer cells such as the cytokines, Interleukin-10 (IL-10) and Transforming Growth Factor Beta (TGF-β), as well as by physical properties of the TME, such as hypoxia, and the extracellular matrix (ECM) [10,11,12]. For example, Shahrzad and colleagues demonstrated that hypoxia promotes global hypomethylation in both cancer and stromal cells [13]. On the other hand, infiltrating immune cells apply pressure on cancer cells to employ strategies to evade the immune system [14]. These mechanisms are regulated by epigenetic changes that allow for dynamic and reversible phenotype switching. Therefore, epigenetics directly affects multiple hallmarks of cancer, including tumor-promoting inflammation and immune evasion. While oncogenic changes are irreversible, epigenetic changes can be modulated to normalize the immune environment and promote cancer immunogenicity, therefore presenting an attractive therapeutic approach supporting immunotherapy.

The combination of immunotherapy with epigenetic drugs that promote immune recognition and responses is a promising new strategy to tackle endocrine-related tumors historically resistant to immunotherapy by providing novel targets for immune recognition, overcoming resistance to checkpoint therapy, and modulating immune cell phenotype and functions.

## 2. Immune Landscape of Endocrine-Related Cancers

**Figure 1:**
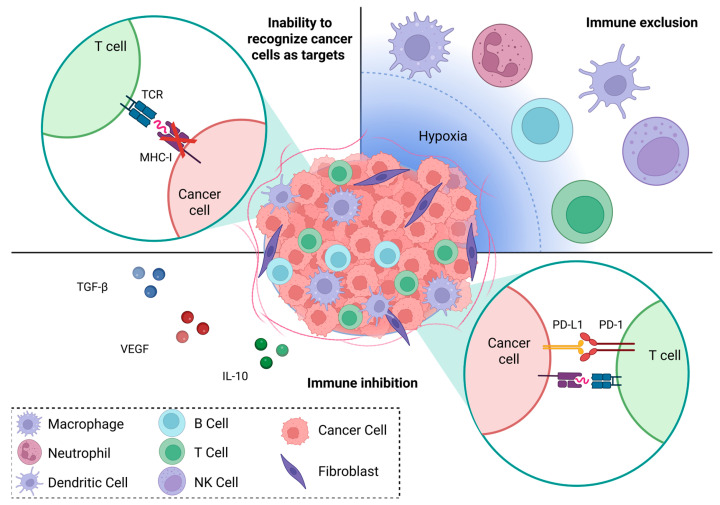
Immune Evasion. Cancers escape elimination by the immune system by evading recognition, selectively excluding immune cells from TME and actively inhibiting immune responses. Created in BioRender. Malecka, A.A. (2025) https://BioRender.com/p3ghanv (accessed on 1 October 2025).

Immune cells can efficiently eradicate cancer. The three main reasons for immune responses to fail are the inability to recognize cancer cells as targets; the inability to reach the cancer site; and finally, the inability to mount an effective immune response (Figure 1). Immunotherapy aims to remove these obstacles.

ERCs are often poorly immunogenic due to a low mutational burden that translates to limited expression of neoantigens, otherwise called tumor-specific antigens (TSAs) [15,16,17,18,19,20,21]. TSAs are present on cancer cells but are absent or weakly expressed in normal tissues, which makes them ideal targets for immunotherapy. TSAs are presented on Major Histocompatibility Complex 1 (MHC-I) molecules, which is necessary for their recognition by activated cancer-specific CD8^+^ T cells [22]. In response to immune pressure, originally immunogenic ERCs can evade recognition by downregulation or loss of TSA or MHC I expression. This results in poor immune infiltration and ineffective immune response. This is further exacerbated by environmental conditions such as hypoxia, which promotes immune exclusion by inhibition of migration and activation of proinflammatory immune cells such as dendritic cells and T cells [23].

Upon reaching ERCs, immune cells encounter a highly anti-inflammatory TME created via multiple mechanisms including secretion of anti-inflammatory cytokines including TGF-β, IL-10, Vascular Endothelial Growth Factor (VEGF) and expression of inhibitory molecules such as Programmed Death—Ligand 1 and 2 (PD-L1/2), and others [24]. These promote phenotypic changes in infiltrating immune cells towards an anti-inflammatory cancer-promoting phenotype. As a result, ERC’s TME is populated by immunoregulatory cells such as tumor-associated macrophages (TAMs), neutrophils, myeloid derived suppressor cells (MDSCs) and regulatory T cells (Tregs) which actively inhibit immune responses [19,25,26,27]. Macrophages are highly plastic cells which can be polarized towards proinflammatory, tumor-suppressive “M1” or anti-inflammatory, tumor-promoting “M2” phenotypes depending on the cues they receive from their environment [28]. The majority of TAMs display M2 phenotypes and correlate with worse prognosis due to inhibition of immune responses and support of cancer cell survival, proliferation and metastasis [29,30,31,32]. However, in some cases, subpopulations of TAMs can promote immune responses and correlate with better patient survival. For example, FOLR2^+^ macrophage population in breast cancer efficiently primes effector CD8^+^ T cells and correlate with better survival [33]. This demonstrates the opportunity to promote immune responses via the modulation of TAM phenotypes. Similar to macrophages, MDSCs are a diverse group of immature myeloid cells, such as neutrophils and monocytes, which are activated under pathologic, anti-inflammatory conditions [34]. MDSCs and TAMs are one of the most numerous immune cells in the ERC TME. Both TAMs and MDSCs inhibit T cell, dendritic cell and NK cell activation, drive T cell exhaustion and promote the recruitment and proliferation of Tregs [35]. With chronic stimulation and upon signaling from surrounding cells, tumor-infiltrating lymphocytes (TILs) become increasingly dysfunctional, acquire an exhausted phenotype and start expressing inhibitory receptors such as PD-1 [36]. The ERC TME therefore contains multiple immune–inhibitory populations acting synergistically, inhibiting the infiltration of the proinflammatory immune population and actively modulating immune phenotypes. For example, prostate cancer is characterized by increased PD-L1 expression and low immune infiltration with elevated numbers of Tregs and MDSC populations [37].

Immunotherapies such as immune checkpoint inhibitors (ICI) aim to reverse this anti-inflammatory state. The expression of PD-L1 and other checkpoint molecules in ERCs correlates with aggressive behavior and occurrence and helps cancers inhibit immune responses [38]. In papillary thyroid carcinoma (PTC), the expression of PD-L1 positively correlated with numbers of TIL and Programmed cell death protein 1 (PD-1) expression, promoting T cell exhaustion and inhibition [39]. Therefore, even immunogenic ERCs such as triple-negative breast cancer (TNBC) can evolve to evade immune recognition leading to primary or acquired resistance in response to immune sculpting [40]. However, the expression of PD-L1 and other checkpoint molecules in prostate cancer and other ERCs does not always correlate with poor prognosis, and the responses of ERCs to checkpoint inhibitors are low [41,42,43,44,45]. This demonstrates the complexity of immune interactions and regulation within TME, as well as the multitude of signals and cells shaping immune responses. The ERCs also have a lower percentage of tumors, with expression of PD-L1 ≥1%, compared to tumors such as lung cancer, for which poorer responses to checkpoint blockade are predicted [46].

The immune TME of ERCs is further complicated by their hormonal landscape. The interactions between hormones and immune responses are complex and context-dependent. Due to local synthesis, estrogen concentration in breast cancer can be 8-fold higher than in plasma [47]. In ovarian cancer, hormonal fluctuation connected with the reproductive cycle results in temporary shifts in local immunity [48]. Estrogen and progesterone have largely opposite effects on the immune system. Estrogen increases the level of PD-1, PD-L1, and other checkpoint molecules in cancers, including ovarian cancer, which reduces the effectiveness of ICIs targeting the PD-1/PD-L1 pathway. At the same time, estrogen promotes the secretion of proinflammatory cytokines and chemokines promoting a chronic inflammatory environment thereby supporting tumor proliferation and migration. On the other hand, progesterone inhibits inflammation and stimulates the expansion of Tregs and M2 TAMs [48]. In a similar way, thyroid hormones T3 and T4 influence the functions and phenotypes of immune and cancer cells. While T4 stimulates PD-1 and PD-L1 expression in cancer cells, studies found that T3 can promote NK cells and M1 macrophage activity [49,50]. In prostate cancer, androgen receptor signaling suppresses MHC class I expression [37]. Therefore, hormones actively and dynamically shape the immune TME, and their interplay with immune regulation can hinder immune therapies. The composition of immune TME affects tumor growth as well as therapeutic responses. TILs have been demonstrated as an important prognostic marker for the majority of tumors, including ERCs. TILs have been found in about half of OC patients and their presence correlates with overall survival [51,52]. Similarly, in prostate cancer, the infiltration of CD8^+^ T cells and T helper 1 cells (Th1) correlates with better survival, while TAMs and Tregs correlate with tumor growth [53]. In BC the value of TILs as predictive markers differs depending on their subtype. While TILs correlate with better response to immunotherapy in HER^+^ and TNBCs, there is no correlation in ER^+^ patients. This is probably due to low level of TILs in ER^+^ patients [54].

Various approaches have been trialed for immunotherapy, so far with limited success. While single-agent ICI is not effective in ERC treatment, the combination of immunotherapy with other therapy types showed some promise, especially in breast cancer. The incorporation of ICI Pembrolizumab with chemotherapy in selected TNBC patients significantly improved patient outcomes [55]. The combination of immuno- and chemotherapy also showed promise in other types of breast cancer [55]. Targeting multiple immune disturbances by combining multiple immune modulators such as ICI with MDSC depletion or tumor vaccine therapy has also been trialed for ERCs. For example, both approaches are currently undergoing clinical trials for prostate cancer, albeit with modest effects [56]. MDSC depleting Cabozantinib in combination with PD-L1 inhibitor Atezolizumab improved progression-free survival (PFS) but not overall survival (OS) [57]. Chimeric Antigen Receptor-T cell (CAR-T) therapy targeting prostate cancer antigens demonstrated an effect only in a subset of patients [56]. Similar modest results are seen in other ERCs, such as ovarian and thyroid cancer [58,59,60].

Modulations of immune TME are largely reversible and driven by epigenetic changes. Epigenetic mechanisms can regulate the phenotype of immune cells as well as immune components of TME. Overall, novel multimodal approaches are required to improve immunotherapy responses, including epigenetic changes, to reverse T cell exhaustion, depleting myeloid cells in the tumor microenvironment and inducing immune cell differentiation to mature effector cells.

## 3. The Rise of Epi-Drugs

Epigenetic regulation of DNA transcription controls cancer initiation, progression and therapeutic resistance [61]. The main mechanisms of epigenetic dysregulation in cancer include aberrant DNA methylation, histone modifications such as acetylation, methylation and phosphorylation, and non-coding RNAs (ncRNA) [62]. DNA methylation describes the transfer of methyl groups to cytosine within CpG dinucleotides, which are catalyzed by DNA Methyltransferases (DNMTs) [62]. Most cancers, including ERCs, are characterized by global hypomethylation, which promotes the expression of oncogenes, and local hypermethylation silencing tumor-suppression genes [63,64,65,66]. Histone acetylation controlled by histone acetyltransferases (HATs) leads to a condensed chromatin structure that promotes gene transcription, while deacetylation results in relaxed chromatin and inhibition of gene transcription and is regulated by histone deacetylases (HDAC) [62]. Differential expressions of HDAC have been observed in ERCs and correspond with disease progression, including the promotion of cancer proliferation and migration and poor clinical outcomes [67,68,69,70,71]. Histone methyltransferases (HMTs) such as Disruptor of Telomeric silencing 1-Like (DOT1L) (methylation of histone H3 lysine 79 (H3K79)) and Enhancer of Zeste homolog 2 (EZH2) (trimethylation of histone H3 lysine 27 (H3K27me3)) are required for histone methylation at lysine and arginine residues. Histone demethylation is processed by enzymes such as lysine-specific demethylases (LSDs.) NcRNAs include the control transcription and post-translational modifications of long non-coding RNAs (lncRNAs).

Given the range of epigenetic modifications that drive cancer etiology, progression and treatment resistance, several drugs have been developed to target these mechanisms. The first approved epi-drug was a DNMT inhibitor (DNMTi), Azacitidine, which established its place in myeloid dysplastic syndrome, whereby the rationale was to induce cell differentiation by reversing hypermethylation and silencing of hematopoietic differentiation genes [72]. In a pivotal, randomized phase 3 trial of Azacitidine versus best supportive care, responses were seen in 60% of patients, with improved quality of life, delay in leukemic transformation and improved overall survival [73]. This led to approval of Azacitidine use for myeloid dysplastic syndrome in 2004. Since then, multiple other epi-drugs have been approved for clinical use. However, the greatest success of epi-drugs so far has been in hematological cancers, with a couple of exceptions observed in solid-organ malignancies. Epigenetic drugs alone or in combination with other therapies have been trialed for various solid cancers, including ERCs (reviewed in the ref. [40]), albeit with limited success due to lack of significant improvement in survival and/or toxicity [74]. Limited success has been reported for a combination of epi-drugs with hormone therapies, particularly in breast cancer. Recently, Tucidinostat (also known as Chidamide), a selective HDAC1, 2, 3, and 10 inhibitor, has been trialed in combination with Exemestane in advanced hormone receptor-positive (HR^+^) breast cancer patients to combat acquired resistance [71,75]. This demonstrated improved PFS in a randomized, phase 3, multicenter trial in China, leading to approval in that country. Disappointingly, longer follow-up demonstrated no improvement in OS over monotherapy [71,75]. Entinostat another HDAC inhibitor targeting HDAC1, 2, and 3 coupled with Exemestane in postmenopausal women with ER^+^ advanced breast cancer demonstrated good safety and promising results in the ENCORE301 randomized phase II study, with increased PFS and OS [76]. This led to a breakthrough therapy approval designation in the US. Again, a subsequent phase III E2112 trial failed to replicate the positive results with no significant improvement in OS upon addition of Entinostat [77]. Similarly, HDAC inhibitors Vorinostat, Pracinostat, Panobinostat, and Romidepsin failed to produce significant improvements in prostate cancer patients and did not move to a phase III trial [78].

Epigenetic drugs and clinical trials in endocrine cancers were recently reviewed by Varun et al. [79]. Interestingly, many therapies including epi-drugs had an effect on immune responses or cells. In the ENCORE301 study, acetylation of peripheral blood mononuclear cells was observed in a subset of patients receiving Entinostat and was correlated with improved outcomes [76]. As current epi-drugs are not selective, they act not only on cancer cells but also on immune cells; therefore, they have the potential to act as immunomodulators enhancing the effects of immunotherapy.

## 4. Epigenetic Modifications Affecting Immune Responses in Cancer

Epigenetic modifications of anti-cancer immune response shape cancer development and therapeutic resistance. Below is an overview of the main epigenetic mechanisms shaping immune responses.

### 4.1. Immune Recognition

**Figure 2:**
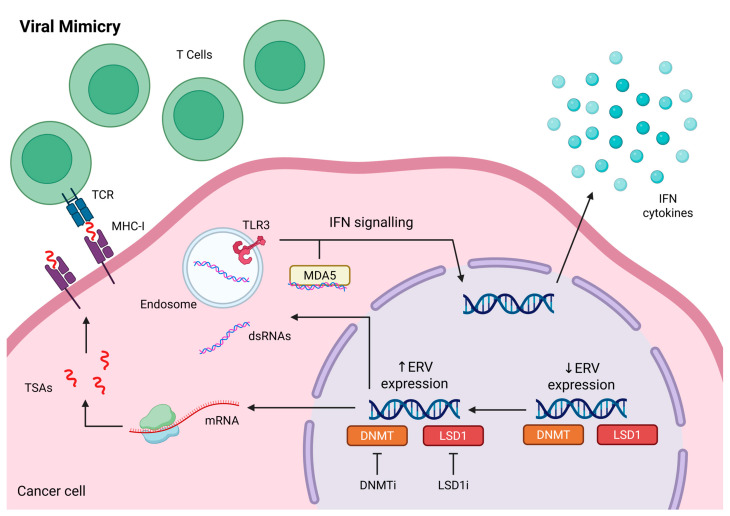
Viral Mimicry. DNMT and LSD1 activity suppresses endogenous retrovirus (ERV) expression. Pharmacological inhibition using DNMTi and LSD1i restores ERV expression, leading to mRNA translation at the ribosome and production of tumor-specific antigens (TSAs). These TSAs are presented on MHC class I molecules for recognition by T cells via TCR. ERV expression also generates double-stranded RNA (dsRNA), which accumulates in the cytoplasm and activates canonical interferon (IFN) signaling through endosomal TLR3 or cytosolic MDA5, ultimately inducing IFN cytokine secretion. Created in BioRender. Malecka, A.A. (2025) https://BioRender.com/ujcazna (accessed on 1 October 2025).

Whilst ERCs exhibit characteristically low mutational load, their global DNA hypomethylation and other epigenetic modifications observed in cancer cells can lead to the expression of TSAs, such as transposable elements (TEs). TEs are short DNA fragments which can move within the genome and include retrotransposons (Class I), such as endogenous retroviruses (ERVs), and DNA transposons (Class II) [80]. ERVs were found to be overexpressed in ERCs, including in breast, ovarian, prostate, and thyroid cancer [81,82,83,84,85]. The expression of ERVs can lead to the formation of double-stranded RNAs (dsRNAs) in the cytoplasm of cancer cells, which trigger the canonical IFN signaling pathway in a process called ‘viral mimicry’ (Figure 2) [86]. Due to the lack or low level of their expression on normal tissues and high immunogenicity, TEs present a very attractive target for the development of cancer vaccines or T cell-based therapies. As Panda et al. demonstrated, high expression of ERVs in different types of cancers, including breast cancer, stimulated immune responses and correlated with increased immunogenicity and immune infiltration with higher CD8^+^ T cell fraction, as well as better responses to PD-1/PD-L1 blockade [87,88]. However, the expression of TEs as well as other TSAs is often heterogenous within the cancer tissue and present only in a subset of patients. Furthermore, in response to immune recognition, cancers employ various epigenetic mechanisms to inhibit ERV expression [89]. For example, Sheng et al. demonstrated that histone H3K4 demethylase LSD1 (KDM1A) downregulates the expression of several ERVs in different cancer cell lines, including breast T47D [89]. Similar observations were made in ovarian cancer, where LSD1 inhibition with SP-2577 promoted the IFNγ pathway driving anti-tumor immunity and T cell infiltration [90]. Epi-drugs can stimulate strong uniform expression of antigens, improving the efficiency of cancer vaccines. For example, Azacitidine can induce ERV transcription, as has been shown by Chiapinelli et al. in ovarian cancer cells, resulting in stimulation of type I interferon response. Inhibition of LSD1 with catalytic inhibitor, GSK-LSD1, increases ERV expression, promoting IFNγ pathway, T cell infiltration and response to anti-PD-1 therapy [89].

Other TSAs, including class II TEs, are also overexpressed in ERCs. For example, the melanoma antigen gene (MAGE) proteins are highly expressed in many tumors, such as breast, prostate and ovarian cancer, due to hypomethylation and other epigenetic mechanisms [91,92,93]. While MAGE expression has been linked to increased cell motility, resistance to cell death, and tumor-promoting inflammation, this intracellular protein is processed by the proteosome into peptides, which are presented by MHC class I on the cancer cell surface, making them a target for T cells [94]. The expression of MAGE, as well as other TSAs including New York Esophageal squamous cell carcinoma 1 protein (NY-ESO-1), has been induced by DNMTi, such as Decitabine (5-Aza-2′-Deoxycytidine), in ovarian and other cancers [94,95,96]. Similarly, NY-ESO-1 ovarian cancers exhibit aggressive phenotypes, poor clinical outcomes, and shorter survival times, yet remain highly immunogenic [97,98,99,100,101]. Induction of NY-ESO-1 by Decitabine alongside NY-ESO-1 vaccine potentiated the effect of the vaccine in ovarian cancer patients [98]. Azacitidine and Decitabine also stimulated NY-ESO-1 expression on thyroid cancer cell lines [102]. However, caution must be exercised when stimulating expression of factors which not only stimulate immune responses but also promote tumor growth and metastasis to ensure the immune system has the capability to eradicate target cells. Inadequate immune responses may lead to immune escape and acceleration of disease progress. Consequently, a careful examination of the individual immune landscape and targeted selection of patients may be necessary.

Cancer cells may also avoid recognition by CD8^+^ T cells via downregulation or loss of antigens or MHC class I protein. Epigenetic mechanisms including DNA methylation and histone acetylation can control MHC class I expression via regulation of its transcription; antigen processing; and presenting machinery components, Beta-2-microglobulin (β_2_M), or MHC-I regulatory proteins. DNA methylation leads to MHC class I downregulation or loss and has been recognized in numerous cancers, including breast, ovarian and thyroid, and can be reversed by hypomethylating agents [103,104,105]. Modulation of ERC immunogenicity by the induction of TSA expression and recognition has wider effects, leading to the global rearrangement of TME immune population. For example, DNMTi Guadecitabine in the presence of IFNγ upregulated MHC class I expression in murine breast cancer while at the same time reducing the proportion of MDSCs and shifting the phenotype of remaining MDSCs into an antigen-presenting phenotype and promoting T cell responses [106,107].

### 4.2. Immune Exclusion

Effective immunotherapy requires immune cells to be able to infiltrate cancer. The number and phenotype of tumor-infiltrating CD8^+^ T cells are one of the best predictors of response to immunotherapy [108]. Cancers employ a variety of mechanisms to inhibit immune infiltration. Peng et al. reported that EZH2-mediated H3K27me3 and DNMT1-mediated DNA methylation repress the secretion of key chemokines mediating T cell influx: C-X-C motif chemokine ligand 9 and 10 (CXCL9/10). Treatment of mice with EZH2 inhibitor, 3-Deazaneplanocin A (DZNep) or DAC reversed this repression of CXCL9 and CXCL10 and improved responses to CD8^+^ T cell therapy in an ovarian cancer model [109]. In breast cancer, including TNBC, LSD1 has been identified as a repressive element for C-C motif chemokine ligand 5 (CCL5), CXCL9 and CXCL10 secretion. LSD1 inhibitors, such as HCI-2509 and Tranylcypromine, significantly increased the expression of these chemokines, thus promoting CD8^+^ influx while also increasing PD-L1 expression [108]. In agreement with these, Ji et al. observed increased infiltration of CD8^+^ T cells with a higher CD8^+^ T cell to Treg ratio in chemoresistant TNBC upon LSD1 inhibition [110]. In early-stage ovarian cancer, the overexpression of the Switch/sucrose non-fermentable-related matrix-associated actin-dependent regulator of chromatin subfamily E member 1 (SMARCE1), which is a gene involved in chromatin remodeling, correlated with higher infiltration of CD8^+^ T cells through increases in CXCL9 and CCL4. Therefore, epigenetic therapy can overcome immune exclusion, increasing the effectiveness of immunotherapy [111].

### 4.3. Immune Regulation—Innate Immunity

Upon entering TME, immune cells encounter a highly immunomodulatory environment promoting an anti-inflammatory phenotype through epigenetic reprogramming [112,113]. In prostate cancer, LSD1 promotes VEGF-A expression, which, in addition to promoting angiogenesis, also has a strong anti-inflammatory effect, promoting M2 macrophages [114]. TGF-β and Colony Stimulating Factor 1 (CSF-1) present in breast cancer trigger epigenetic modulation of the TAM phenotype to promote tumor growth [112]. The involvement of epigenetic machinery in controlling the TAM phenotype presents an attractive therapeutic target. In ovarian cancer, treatment with Azacitidine in the mouse model DNMTi led to an increase in the M1/M2 macrophage ratio as well as recruitment of other immune cells, such as CD8^+^ T cells and NK cells, while also decreasing MDSC levels [115]. DNMTi Guadecitabine is another epi-drug with a wide-ranging effect on the tumor immune microenvironment, reducing and phenotypically altering MDSC, as shown in breast cancer [106]. Entinostat has shown promising results, supporting checkpoint therapy through altering MDSC functions and reducing the production of Arginase 1 and expression of PD-L1 [35]. In a randomized phase 2 trial, Entinostat showed a reduction in MDSC, decreased CD40 in MDSC and increased MHC Class II (HLA-DR) expression on circulating monocytes, confirming its immunomodulatory effect [76,116]. While histone modifications are a promising therapeutic strategy for determining MDSC accumulation and phenotype, several challenges remain to be addressed, such as dosage, timing, and drug combination. For example, while a low dose of Trichostatin A reduces MDSC recruitment, a high dose can paradoxically increase recruitment and promote an anti-inflammatory phenotype of MDSCs [113].

### 4.4. Adaptive Immunity—Checkpoint Inhibitors and Exhausted T Cells

Epigenetic modulation of cancer immunogenicity and immune cell phenotypes affect adaptive immune responses. Stone et al. demonstrated in a mouse model of epithelial ovarian cancer that Azacitidine-induced reduction in TAMs and MDSCs was associated with increased numbers of NK cells and CD8^+^ T cells [117]. Furthermore, a double combination of DNMTi and HDACi enhanced the response to checkpoint inhibitor α-PD-1 [117].

However, epigenetic regulation also affects TILs, directly driving their differentiation towards exhaustion impacting the efficiency of checkpoint blockade inhibitors, cell therapy and cancer vaccines. Temporal analysis of TIL exhaustion in TME uncovered two distinct phases of epigenetic modification, driving the exhausted phenotype [118]. The initial (early) phase, which was reversible, was followed by a final irreversible phase, leading to the acquisition of a terminally exhausted phenotype. While both phases were characterized by extensive chromatin modifications, the sites of these modifications differed [118]. The epigenetic regulation of T cell exhaustion has important implications for immune therapies aiming to reinvigorate the immune environment within TME and reverse the exhausted T cell phenotype, such as T cell transfer therapies, for example, CAR-T cells. Epi-drugs such as class I HDACi, M344, and Chidamide can improve the memory of CAR-T cells and promote resistance to terminal exhaustion in a B cell acute lymphoblastic leukemia xenograft model [119]. Interestingly, M344 was also shown to suppress breast cancer cell proliferation demonstrating the ability of epi-drugs simultaneously target multiple cellular components of the TME [120].

Cancer drives T cell dysfunction and exhaustion by targeting checkpoint receptors such as PD-1/2 and LAG3. Epigenetic regulation drives the expression of checkpoint ligands in cancer cells and infiltrating immune cells, such as TAMs, in response to T cell infiltration and IFNg secretion [121,122]. PD-1 is a key inhibitory receptor expressed in leukocytes and is a common target of cancer immune inhibition. For example, in breast cancer, the expressions of METTL2 and lncRNA Metastasis-associated lung adenocarcinoma transcript 1 (Malat-1) mediate PD-L1 expression, while in PTC, DNA methylation inversely correlates with the expression of checkpoint molecules [116,120,121]. Multiple epigenetic mechanisms, including histone acetylation, methylation, and microRNAs (miR), have been shown to regulate PD-L1 expression in breast cancer cells lines [123,124,125,126]. LSD1 is overexpressed in several cancers, including breast, prostate, ovarian and thyroid cancer, promoting cancer cell proliferation and migration [114,127,128,129,130]. In T cells, LSD1 downregulates PD-1 expression [131]. However, LSD1 inhibition with HCI-2509 upregulates PD-L1 expression in breast cancer, while SP-2577 exerts a similar effect in ovarian cancer [90,108]. Class I HDACi increase PD-L1 expression in prostate cancer [132]. MiRs play regulatory roles in PD-L1 expression. MiR-200 inhibits PD-L1 upregulation in ovarian cancer [133]. Cancer cells can also promote PD-L1 expression in TAMs through miR presentation via extracellular vesicles containing miR-27a-3p, as demonstrated in breast cancer [134]. In pancreatic cancer, miR-93 and miR-106b inhibit PD-L1 expression [135]. LncRNAs are also involved in PD-L1 regulation in ERCs. For example, lncRNA GATA binding protein 3-antisense 1 (GATA3-AS1) stabilizes PD-L1 expression in TNBC [136]. LncRNA HOXA transcript at the distal tip (HOTTIP) facilitates ovarian cancer immune escape via increasing IL-6, which promotes PD-L1 expression in neutrophils [137]. While the majority of research focuses on the PD1/PD-L1/2 pathway, other immune regulatory receptors are also modified via epigenetic regulation. For example, in chemoresistant ovarian cancer, histone acetylation and accumulation of DNA methylation correlate with suppressed expression of the 4-1BB ligand (4-1BBL/CD157) and OX-40 ligand (OX-40L/CD252) [138]. The 4-1BBL and OX-40 ligand promote cytotoxic T cell responses [138].

### 4.5. Immune–Epigenetic–Hormonal Axis in ERCs

The progress of ERCs is influenced by the interplay between hormonal, epigenetic and immune mechanisms, which are intrinsically and reciprocally connected.

Hormonal signaling directly regulates immune cell phenotypes via epigenetic mechanisms. Estrogen promotes anti-inflammatory macrophage phenotypes via induction of DNMT1, which inhibits p53 expression [139]. On the other hand, progesterone can attenuate LPS-induced cytokine production in murine macrophages and dendritic cells [140,141]. While the immunomodulatory effect of thyroid hormones on immune cells’ epigenome is less studied, Shepherd et al. demonstrated changes in DNA methylation in human monocytes in response to T3 [142]. Immune cells can also influence the expression of hormone receptors and sensitize cancer cells to estrogen indirectly via epigenetic mechanisms. Macrophages induced ER expression in endometrial cancer cells by secreting IL-17, which modulated ten-eleven-translocation 5-methylcytosine dioxygenase (TET1) [143]. Therefore, hormonal signaling in ERCs not only influences immune responses via epigenetic modification but may also promote therapeutic resistance.

Hormones bind to their respective receptors, whose expression and function are regulated by multiple mechanisms including epigenetics [144]. Epigenetic dysregulation such as DNA methylation was shown to affect the expression of androgen, estrogen and progesterone receptors in various ERCs, including breast, prostate and ovarian cancers [145,146,147,148,149,150]. The expression of thyroid-stimulating hormone receptor (TSHR) is also found to be silenced due to DNA methylation in epithelial thyroid cancers [151]. Zhou et al. demonstrated the ability of epi-drugs to restore ER expression in ER-negative human breast cancer lines, MDA-MB-231 and MDA-MB-435 [152]. On the other hand, Kondo et al. demonstrated that miR-206 can downregulate ERα in human MCF-7 breast cancer cell lines [153]. In reciprocal interactions, hormones affect the expression of epigenetic-modifying genes, as reviewed by Zhang and Ho [144]. 

The interplay between hormones, epigenetic mechanisms, and the immune environment plays an important role in the regulation of cancer growth and anti-cancer immune responses, and further research is needed to identify their roles in therapeutic resistance.

## 5. Targeting Epigenetic Dysregulation to Overcome Immunotherapy Resistance

The progress in understanding how epigenetic dysregulation affects immunity in ERCs supports the rationale for combining epi-drugs with immunotherapy to overcome resistance. Pre-clinical studies are promising; for example, in a mouse model of ovarian cancer, the combination of DNMTi Decitabine with anti-CTLA-4 increased the recruitment of NK cells and CD8^+^ T cells and prolonged survival [154]. Entinostat synergized with an IL-15 superagonist and vaccine in murine breast cancer model [155]. The combination enhanced infiltration and activation of T cells with concomitant reduction in Tregs [155].

While preclinical models suggest the value of epigenetic priming for immunotherapy and the ability of epi-drugs to turn a “cold” tumor into a “hot” tumor, clinical trials point to a more complex picture. Several clinical trials in ERCs using a combination of epi-drug with immunotherapy have been completed or are currently in progress with results expected soon (Table 1). The majority of trials combine DNMT and HDAC inhibitors with ICI for breast and ovarian cancers. Breast, ovarian, and colorectal cancer patients treated with a combination of DNMTi oral Azacitidine and PD-L1 inhibitor, Durvalumab (NCT02811497), did not show clinical responses, even when the protocol was amended to add vitamin C [156]. The rationale behind adding vitamin C was based on its ability to promote demethylation, thereby potentially enhancing the effect of Azacitidine. The lack of clinical response may be due to low methylation levels, which were not able to increase tumor immunogenicity. Low methylation levels were detected in PBMCs indicating the systemic activity of CC-486; however, there was no effect at the tumor site. Authors speculated that the oral form of Azacitidine used in this study has poor penetration or demethylation activity in the tissue. Another possibility is that higher hypomethylation is required to turn cold tumors into hot tumors in patients with later stages of the disease who have already had multiple rounds of treatment. This trial demonstrates the difficulty in translating preclinical studies into clinical protocols. Encouraging results were reported from an ovarian cancer trial (NCT01673217) combining DNMTi Decitabine with NY-ESO-1 vaccine and Doxorubicin [98]. Disease stabilization or partial response was observed in 6 out of 10 patients. The response was associated with antigen spreading, which may be due to the effect of Decitabine; however, further studies in this area are required. Two other studies investigated the effect of HDACi Entinostat with PD-L1 inhibitors, Atezolizumab in breast cancer patients (NCT02708680, ENCORE 602, TRIO025) and Avelumab in ovarian cancer patients (NCT02915523). In both trials, there was no improvement seen over monotherapy with ICI only. In the case of ENCORE trial, the combination resulted in greater toxicity [157]. Due to the lack of specificity of epi-drugs and differences in immune composition of the TME in individual patients, the safety of combination therapy must be carefully considered. Further research into biomarkers and optimal immune TME is required to improve therapeutic efficacy and ensure patient safety.

Promising results were observed in a breast cancer trial (NCT02453620), where Entinostat was combined with Nivolumab (PD1 blocking antibody) and Ipilumab (CTLA-4 blocking antibody) [158]. The clinical benefit rate reported from this study reached 40%. This combination therapy promoted immune responses, including M1 TAMs, pDC, NK cells, and T helper 1 cells. Gene expression analysis revealed significant differences in the cellular composition of immune TME between responders and non-responders. Responders demonstrated basal enrichment in immune-related pathways and genes associated with anti-tumor immune responses [158]. Stratifying patients based on immune TMA composition and evidence of anti-tumor immune responses may be necessary to deliver appropriate therapeutic regimes. Further research into identifying suitable biomarkers is necessary.

HDACi and Vorinostat combined with Pembrolizumab (PD1 blocking antibody) (NCT02619253) has been investigated in prostate cancer patients in a phase I trial. Responses were observed in 2 out of 12 patients (response rate 17%). Both Vorinostat and Pembrolizumab showed some activity in prostate cancer patients; therefore, it is not known if the combination has an advantage over monotherapy [159]. However, the study suggested that lower levels of mononuclear MDSCs in peripheral blood correlated with clinical benefit in patients receiving combination therapy [159]. Identifying potential responders is crucial to be able to deliver effective therapy in the future. Pembrolizumab in combination with DNMTi Guadecitabine (NCT02901899) demonstrated promising results in platinum-resistant ovarian cancer patients. In total, 8 out of 35 patients had stable disease and 3 partial responses were observed, resulting in a clinical benefit rate of 31.4%. Responders’ peripheral blood mononuclear cells showed post-treatment hypomethylation with increased frequency of CD4^+^ T cells and classical monocytes, therefore suggesting effective epigenetic priming [160]. The authors investigated predictive markers for ICIs in ovarian cancer patients and suggested the presence of activated classical monocytes, naïve and/or central memory CD4^+^ T cells, as well as a higher density of CD8^+^ T cells and CD20^+^ B cells and the presence of tertiary lymphoid structures in tumors, associated with durable responses. More clinical trials are currently underway. Epigenetic drugs have the ability to promote immune responses and, at the same time, inhibit the survival, proliferation and migration of cancer cells.

**Table 1:** Selected clinical trials combining epigenetic and immunotherapy for ERCs.

ERC Type	NCT Number	Epi-Drug	Epigenetic Target	Immunotherapy	Immune Target	Other Intervention	Status	Phase	References
prostate	NCT04388852	DS3201	EZH1/2 inhibitor	Ipilimumab	CTLA-4		active	1	
prostate	NCT02619253	Vorinostat	HDACi	Pembrolizumab	PD-1		completed	1	[159,161]
ovarian	NCT01673217	Decitabine	DNMTi	NY-ESO-1 vaccination	NY-ESO-1 antigen	doxorubicin	completed	1	[98]
ovarian	NCT02901899	Guadecitabine	DNMTi	Pembrolizumab	PD-1		completed	2	[160]
ovarian, breast	NCT02811497	CC-486 (oral azacitidine)	DNMTi	Durvalumab	PD-L1	+/− vitamin C	completed	2	[156]
ovarian	NCT02900560	Azacitidine	DNMTi	Pembrolizumab	PD-1		terminated	2	[162]
ovarian	NCT03206047	Guadecitabine	DNMTi	Atezolizumab +/− NY-ESO targeting vaccine (CDX-1401)	PD-L1 +/− NY-ESO-1 antigen		completed	1,2	
ovarian, breast	NCT03292172	RO6870810	BETi	atezolizumab +/− NY-ESO targeting vaccine (CDX-1401)	PD-L1 +/− NY-ESO-1 antigen		terminated	1	[163]
ovarian	NCT04840589	ZEN-3694	BETi	Nivolumab +/− Ipilimumab	PD-1 +/− CTLA-4		recruiting	2	
ovarian	NCT02915523	Entinostat	HDACi	Avelumab	PD-L1		completed	1,2	[164]
breast	NCT02453620	Entinostat	HDACi	Nivolumab + ipilimumab	PD-1 + CTLA-4		active, not recruiting	1,2	[158,165]
breast	NCT02708680	Entinostat	HDACi	atezolizumab +/− NY-ESO targeting vaccine (CDX-1401)	PD-L1		completed	1,2	[157]
breast	NCT02395627	Vorinostat	HDACi	Pembrolizumab	PD-1	anti-estrogen (tamoxifen)	terminated	2	[166]
breast	NCT02393794	Romidepsin	HDACi	Nivolumab	PD-1	cisplatin	unknown/completion estimated 2025	1,2	

### Challenges in Designing Epigenetic and Immunotherapy Combination Strategies for ERCs

Combination therapy of epigenetic modification and immunotherapy provides an attractive novel approach for targeting treatment-resistant ERCs. However, important challenges currently impact the efficacy and safety of this approach. These challenges include (1) the lack of molecular and cellular specificity of epi-drugs; (2) the hormonal–epigenetic–immune interplay; (3) the lack of biomarkers; (4) multicellular interactions; (5) dose dependent effect of epi-drugs; (6) drug formulation and effective targeted delivery; and (7) the effect of TME on therapeutic responses and patient heterogeneity in the immune landscape.

The lack of target specificity of epi-drugs and the complex multicellular interactions within cancer environments are both opportunities and significant challenges to the design of epigenetic therapy. Epi-drugs target cancer cells, and various cells present in the TME distinctly affect various subsets of cells. Currently available inhibitors alter multiple genes simultaneously in various cell types making it difficult to predict the overall effect in complex multicellular systems. Therefore, depending on the composition, immune TME epi-drugs may have a beneficial or detrimental effect. DOT1L is responsible for H3K79 methylation and is often upregulated in BC [167]. DOT1L inhibition silences the expression of ERα gene and in effect inhibits the proliferation of antiestrogen-resistant BC cells. This makes DOTL1 an attractive target for endocrine-therapy-resistant ER^+^BC patients [168]. DOT1L also plays an important role in regulation of innate immunity. Overexpression of DOTL1 in murine colitis models promoted the activation of MDSCs with subsequent inhibition of T cell proliferation [169]. Furthermore, the inhibition of DOTL1 promoted IFNγ production by Th1 cells [170]. The silencing of DOTL1 downregulates the production of IL-6 and IFN-β in activated macrophages with no effect on TNFα production, therefore suggesting a more nuanced modification of immune responses [171]. The role of DOTL1 in the regulation of multiple immune populations such as Tregs and mast cells is less known and requires more research. Interestingly, Yang et al. showed that while targeting DOT1L in hepatoma cells in vitro inhibited their invasive potential. This effect was lost in vivo due to the signaling from infiltrating macrophages, demonstrating the importance of immune TME in regulating therapeutic responses to epi-drugs [172].

The cell type, specific molecular target, dose and formulation also affect the therapeutic outcome. LSD inhibitors promote CD8^+^ T cells responses; however, they can also attenuate NK cell responses [130,173,174,175,176]. However, the repressive effect of LSD1 inhibitors on NK cells was restricted to scaffolding inhibitors (SP-2509 and SP-2577), which disrupt epigenetic complexes that include LSD1, and was not observed for catalytic inhibitors or T cells [174].

The effect of epi-drugs on immune cells can depend on used dose. Rosborough et al. demonstrated that HDACi can increase the number of murine MDSCs in in vitro and in vivo models using Trichostatin A [177]. On the other hand, Vorinostat, another HDACi, was shown to decrease number of MDSCs induced by 4T1 mammary tumors in vivo and in vitro [178]. However, the study used higher doses of HDACi, demonstrating the potential for the dose-dependent effect of epi-drugs. This may lead to poor translation between preclinical and clinical results, which can be further affected by suboptimal drug delivery.

The heterogeneity of patients’ immune landscape and the lack of reliable biomarkers for combination therapy can further hinder the therapy and lead to toxicity and acceleration of tumor growth and spread in some patients. Epi-drugs can be used to induce the expression of highly immunogenic antigens such NY-ESO-1 and MAGE to support cancer vaccines targeting these antigens and invigorate immune responses [94]. However, these antigens correlate with aggressive and invasive tumor phenotypes; therefore, if the immune response cannot eliminate the tumor, it will lead to acceleration of tumor progression. It is therefore paramount to be able to identify suitable candidates for this combination therapy, and more research is still needed in this area. The results from clinical trials demonstrated the importance of epigenetic and immune markers, such as basal methylation levels and enrichment in specific immune signature and patients’ immune landscape. Combination of both immune and epigenetic markers may allow for better stratification of patients. Future research should focus on integrating multi-omic profiling and longitudinal monitoring to refine patient selection and optimize therapeutic outcomes.

## 6. Conclusions

Cancers constantly evolve in response to pressures from the immune system. Epigenetic regulation, which allows for dynamic and reversible modifications of cell characteristics and their environment, plays a key role in tumor immune escape. Therefore, epi-drugs present a promising strategy through their addition to immunotherapy by modulating multiple branches of immune response, as well as the behavior of cancer cells. However, understanding the mechanisms and consequences of combination therapy is paramount for successful combination therapy. For example, while promoting the expression of MAGE and NY-ESO-1, using demethylating agents offers the opportunity to increase tumor immunogenicity and enhanced immune responses, potentially leading to elimination of the tumor. Both antigens correlate with more aggressive cancer phenotype and worse prognosis due to the promotion of tumor proliferation, migration and invasion. Therefore, if the immune response is not strong enough to eliminate cancer cells, the therapy may instead lead to cancer progression. The type of immune response enhanced by epi-drugs is also important. While LSD inhibitors can promote CD8^+^ T cell responses and reduce the growth of cancer cells, they may also lead to the inhibition of NK cell responses [130,173,174,175,176]. The lack of target specificity towards the cell type and genes represents both an advantage and challenge to epigenetic therapy. Current epigenetic drugs exert a global effect on tumor cells and its TME, modulating a large number of genes in multiple targets that require further investigation and validation. Better identification of the targets of epigenetic therapy is necessary, and the understanding of individual immune landscapes is paramount for successful combination and may be the reason for the low success rates of current clinical trials. Finally, as discussed by Tian et al., the dose and drug combination of epi-drugs can modulate their effect [113]. In conclusion, the combination of epigenetic modulation and immunotherapy presents a promising avenue for treating resistant ERCs. However, further research into epigenetic targets and individual tumor landscapes is urgently needed.

## Abbreviations

4-1BBL	4-1BB ligand
Arg-1	Arginase 1
BC	Breast cancer
CAR-T	Chimeric antigen receptor-T cell
CCL4/5	C-C motif chemokine ligand 4/5
CD4/8/40	Cluster of differentiation 4/8/40
CSF-1	Colony stimulating factor 1
CTLA4	Cytotoxic T-lymphocyte-associated protein 4
CXCL9/10	C-X-C motif chemokine ligand 9/10
DNA	Deoxyribonucleic acid
DNMT	DNA methyltransferase
DNMTi	DNA methyltransferase inhibitor
DOT1L	Disruptor of telomeric silencing 1-like
dsRNA	double-stranded RNA
DZNep	3-deazaneplanocin A
ECM	Extracellular matrix
ER	Estrogen receptor
ERC	Endocrine-related cancer
ERV	Endogenous retrovirus
EZH2	Enhancer of Zeste homolog 2
FOLR2	Folate receptor beta
GATA3-AS1	GATA binding protein 3-antisense 1
H3K27me3	Histone H3 lysine 27 trimethylation
H3K4	Histone H3 lysine 4
H3K79	Histone H3 lysine 79
HAT	Histone acetyltransferase
HDAC	Histone deacetylase
HDACi	Histone deacetylase inhibitor
HER	Human epidermal growth factor receptor
HLA-DR	Human leukocyte antigen-DR isotype
HMT	Histone methyltransferase
HOTTIP	Homebox A transcript at the distal tip
HR	Hormone receptor
ICI	Immune checkpoint inhibitor
IFN	Interferon
IFNγ	Interferon gamma
IL-10	Interleukin 10
lncRNA	Long non-coding RNA
LPS	Lipopolysaccharide
LSD	Lysine-specific demethylase
MAGE	Melanoma antigen gene
Malat-1	Metastasis-associated lung adenocarcinoma transcript 1
MDSC	Myeloid derived suppressor cell
METTL2	Methyltransferase 2
MHC-I	Major histocompatibility complex class 1
miR	MicroRNA
ncRNA	Non-coding ribonucleic acid
NK	Natural killer
NY-ESO-1	New York esophageal squamous cell carcinoma 1
OS	Overall survival
OX-40L	OX-40 ligand
PD-1/2	Programmed cell death protein 1/2
pDC	Plasmacytoid dendritic cells
PD-L1/2	Programmed death-ligand 1/2
PFS	Progression-free survival
PTC	Papillary thyroid cancer
RNA	Ribonucleic acid
SMARCE1	Switch/Sucrose non-fermentable-related matrix-associated actin-dependent regulator of chromatin subfamily E member 1
TAM	Tumor-associated macrophage
TE	Transposable element
TET1	Ten-eleven translocation 1
TGF-β	Transforming growth factor beta
Th1	T helper 1
TIL	Tumor-infiltrating lymphocyte
TME	Tumor microenvironment
TNBC	Triple negative breast cancer
Treg	Regulatory T cell
TSA	Tumor-specific antigen
TSHR	Thyroid-stimulating hormone receptor
VEGF	Vascular endothelial growth factor
VEGF-A	Vascular endothelial growth factor A
β2M	Beta-2 microglobulin
